# A prolonged hiatus in postmenopausal HRT, does not nullify the therapy’s positive impact on ageing related sarcopenia

**DOI:** 10.1371/journal.pone.0250813

**Published:** 2021-05-05

**Authors:** Gladys L. Onambélé-Pearson, David J. Tomlinson, Christopher I. Morse, Hans Degens

**Affiliations:** 1 Musculoskeletal Sciences and Sport Medicine Research Centre, Department of Sport and Exercise Science, Manchester Metropolitan University Institute of Sport, Faculty of Science & Engineering, Manchester Metropolitan University, Manchester, United Kingdom; 2 Musculoskeletal Sciences and Sport Medicine Research Centre, Department of Life Sciences, Manchester Metropolitan University, Manchester, United Kingdom; 3 Lithuanian Sports University, Kaunas, Lithuania; Universidad Nacional Autonoma de Mexico, MEXICO

## Abstract

**Background:**

Previous work suggest a positive skeletal muscle effect of hormone replacement therapy (HRT) on skeletal muscle characteristics This study aimed to quantify any continued positive effect of HRT even after a sustained hiatus in treatment, controlling for two key muscle modulation hormones: Estradiol (E2) and Tri-iodo-thyronine (T3).

**Method and findings:**

In 61 untrained women (18-78yrs) stratified as pre-menopausal, post-menopausal without (No_HRT) and post-menopausal with (Used_HRT) HRT history, body composition, physical activity, serum E2 and T3 were assessed by dual energy x-ray absorptiometry, Baecke questionnaire and ELISA. *Gastrocnemius medialis* (GM) and *tibialis anterior* (TA) electromyographic profiles (mean power frequency (mPowerF)), isometric plantar-flexion (PF) and dorsi-flexion (DF) maximum voluntary contraction (MVC), rate of torque development (RTD), isokinetic MVC and muscle volume, were assessed using surface electromyography, dynamometry and ultrasonography. Muscle quality was quantified as MVC per unit muscle size. E2 and E2:T3 ratio were significantly lower in postmenopausal participants, and were positively correlated with RTD even after controlling for adiposity and/or age. Pre-menopausal females had greater MVC in 8/8 PF and 2/5 DF (23.7–98.1%; *P*<0.001–0.049) strength measures compared to No_HRT, but only 6/8 PF (17.4–42.3%; *P*<0.001–0.046) strength measures compared to Used_HRT. Notably, Used_HRT had significant higher MVC in 7 PF MVC (30.0%-37.7%; *P* = 0.006–0.031) measures than No_HRT, while premenopausal and Used_HRT had similar uncorrected muscle size or quality. In addition, this cross-sectional data suggest an annual reduction in GM muscle volume corrected for intra-muscular fat by 1.3% in No_HRT and only 0.5% in Used_HRT.

**Conclusion:**

Even years after cessation of the therapy, a history of HRT is positively associated with negating the expected post-menopausal drop in muscle quantity and quality. Whilst mPowerF did not differ between groups, our work highlights positive associations between RTD against E2 and E2:T3. Notwithstanding our study limitation of single time point for blood sampling, our work is the first to illustrate an HRT attenuation of ageing-related decline in RTD. We infer from these data that high E2, even in the absence of high T3, may help maintain muscle contractile speed and quality. Thus our work is the first to points to markedly larger physiological reserves in women with a past history of HRT.

## Introduction

Animal and human models have long shown that ageing is associated with a substantial slowing in contraction duration and half-relaxation time of skeletal muscle fibers [1]. These functional changes in the twitch parameters are accompanied with an increase in the relative proportion of slow type I fibers compared with fast type II fibers [1–5]. In fact, age-related slowing of contractile properties may well be attributable to a preferential atrophy of fast fibers, resulting in an increased areal percentage of type I fibers [6], and a slowing of type I [7] and type IIa fibers [8]. What is also widely known is that there are over 3000 genes that are differentially expressed in males vs female skeletal muscle [9]. It is also clear that the sex-based differences are regulated by a number of hormones, especially thyroid hormone and gonadal hormones (for a review please read [10].

In much the same way as ageing affects estrogen levels, exercise training is also an important modulator of estrogen levels, thus establishing the efficacy of estrogen in muscle hypertrophy. In previous work, two key observations were made: (1) anabolic androstenedione-doped human participants exhibit increased testosterone and subsequently estrogen levels, (2) anabolic androstenedione-doped human participants show significant increases in lean tissue content [11]. This in itself is not a surprise since the aromatization of testosterone to estrogen, is necessary for the muscle hypertrophic effect of the former to become possible [12]. In fact, work using amenorrheic [13] or post-menopausal females [14], is especially relevant here in demonstrating the theorem that estrogen is important for skeletal muscle structure and function. Indeed a substantial body of work shows a link between lowered circulating estrogen levels, and muscle atrophy as well as poor physical performance [15, 16]. Also of note, is the previous research showing that obesity is one of the key factors linked to an overall increase in concentrations of free circulating biologically active estradiol (17β-estradiol). In fact, such research shows that in overweight/obese women a 16–24% higher circulating level of estradiol is observed compared to age-matched counterparts, even after adjustment for covariates [17]. Also of relevance, is the observation that obesity is associated with a higher proportion of glycolytic type IIB fibers (for a review, see [18]). Thus, it appears important to take body composition (% body fat and % lean tissue) into account when considering the impact of estrogen in modulating the rate of age-related loss of skeletal muscle mass and strength in older women.

As mentioned above, estrogen and thyroid are two key hormones in relation to the origin of sex-based differences in skeletal muscle phenotype. Thyroid hormone, like estrogen, is a pluripotent hormone and it exhibits dual effects in many systems. The link between thyroid hormones and muscle fiber type is an often overlooked relationship especially in *in vivo* studies. Notwithstanding this omission, the body of work suggesting that thyroid levels ought to be considered in any study pertaining to comment on muscle fiber phenotype is substantive. A case in point, hypothyroidism is a clinical condition in which up to 80% of patients experience one or another form of myopathy [19]. Moreover, as far back as the mid-1970s, it was first reported that in humans, hypothyroid myopathy is linked to biopsy-assessed type II fiber type atrophy in the *vastus lateralis* muscle [20]. Corroborating a causal link between fiber type and thyroid hormones, and adding an element of gender specificity to this relationship, previous research directly supplemented male and female rats with tri-iodo-thyronine (T3), and quantified the velocity of muscle contractions as well as myosin heavy chain composition. The researchers report that whilst the T3 treatment increased the velocity of contraction as well as upregulated fast myosin heavy chain (irrespective of age), this effect was not seen in the male models, suggesting an interaction between estrogen and thyroid in the regulation of fiber type composition [21]. Others have also observed a gonadal-hormones-linked sensitivity to T3 effects, at least in animal models [22]. As such it is pertinent that in an effort to individualize tomorrow’s clinical interventions, an understanding of how various factors interact is clarified. A group known to experience drastic deleterious changes in physical function, hence worthy of further study, is post-menopausal women.

Despite this existing body of work, there is no current study clarifying, using non-invasive or otherwise methods, the degree to which gonadal and thyroid hormones influence skeletal muscle phenotype in the postmenopausal female. The objective of the current cross-sectional study was to determine any covariation between thyroid hormone (Tri-iodo-thyronine (T3)) and Estradiol (E2) levels and *in vivo* muscle size, strength, quality and indirect indices of fiber type composition in pre vs. post-menopausal women. A secondary objective was to determine the degree to which HRT history preserves post-menopausal muscle function. Therefore, it was hypothesized that: (1) Lower levels of E2 (and/or no HRT history) is linked to decreased muscle function (decreased isometric MVC, isokinetic strength and muscle quality), (2) Ageing is associated with perceptible decrements in T3 levels thereby modulating muscle activation profile and hence potentially indicating fiber type percentage shift towards a slow phenotype (resulting in slower rate of torque development–RTD—and lower Mean Power Frequency–mPowerF-); (3) estradiol to Tri-iodo-thyronine ratio (i.e. E2:T3) is a useful endocrine modulator of skeletal muscle phenotype, (4) high body fat in the post-menopausal status alters muscle EMG profile and RTD (where both are potential indirect indices of muscle fiber type- see details in the Methods), likely through decreasing the fast-to-slow transition of fiber type) in older women.

## Materials and methods

### Participants

Sixty-one untrained women aged 45 ± 22yrs (range: 18-78yrs) that were considered representative for the wider population were recruited and categorized by their menopause status and additionally by HRT use history (only postmenopausal). Participants were recruited using convenience sampling by word of mouth and presentations at both Manchester Metropolitan University and the University of the Third Age in Cheshire (UK). All studies took place at the laboratories of the Research Centre for Musculoskeletal Science and Sports Medicine (previously Health Exercise & Active Living) at the Cheshire campus of Manchester Metropolitan University. Prior to the undertaking of assessments, a general health questionnaire and screening of participants’ level of physical activity was ascertained using the Baecke physical activity questionnaire that is composed of 3 sections detailing, work, structured sport and leisure physical activity [23]. During this process, participants’ menopausal age, use of HRT and duration of treatment, use of the contraceptive pill and years of use, along with any current medications that may affect thyroid T3 levels were noted. Participants’ were excluded if their current level of exercise exceeded 2 hours of structured moderate/vigorous physical activity per week, if they had substantially changed their habitual physical activity levels and diet in the past 12 months and were taking any medication that may affect maximum effort during testing. Prior to the commencement of the study, participants gave their written informed consent and all the procedures in this study had approval from the local Manchester Metropolitan University ethics committee (Ethics Committee Reference Number: 09.03.11 (ii)).

### Protocol

On the first visit to the laboratory, participants completed the informed consent form and were screened for potential participation using their answers in the general health and physical activity questionnaire. Following acceptance onto the study, participants then undertook a 7-minute dual energy x-ray absorptiometry (DEXA) scan followed by a fasted blood sample and familiarization of the strength testing procedure. On their second and final visit to the laboratory, participants completed the full strength protocol assessing maximal voluntary contraction (MVC), rate of torque development, both muscle activation and co-activation of the *gastrocnemius medialis* (GM) and *tibialis anterior* (TA) muscles using surface electromyography (sEMG), and finally GM muscle volume.

### Measurement of body composition

DEXA (Hologic Discovery: Vertec Scientific Ltd, Reading, UK) was used to ascertain participants’ body composition following an overnight 12-hour fast. A detailed description of the 7-minute standardized scanning procedure (whole body, EF 8.4 lSv) is reported previously [24]. Scan results were calculated using the Hologic APEX software (version 3.3) and presented in terms of whole body lean mass and fat mass, with the same researcher completing scanning and analysis. Primarily DEXA was utilized to quantify total lean and fat mass (along with body fat percentage) for general descriptive characteristics in order to observe how well the sample was matched owing to the influence body composition has upon skeletal muscle characteristics [24]. Secondly, total fat and body fat percentage were utilized as a covariate in correlations due to its impact on estrogen levels [17].

### Serum collection

Following the completion of the DEXA scan, 61 participants provided a 10mL fasted (12 hours) blood sample between 8am and 9am, having not performed strenuous exercise for 48 hours prior. Blood was collected in anticoagulant-free vacutainers (BD Vacutainer Systems, Plymouth, UK) and rested on crushed ice for 10–15 minutes. Samples were then placed into a refrigerated centrifuge at 4°C (IEC CL31R, Thermo Scientific, Massachusetts, United States) for 10 minutes at 2700 ⊆ g after which serum was extracted and stored in 2mL aliquots at −20°C until subsequent analysis.

### Muscle strength

Following familiarization on the first visit to the laboratory to the strength testing procedure, plantar flexion (PF) and dorsiflexion (DF) MVC torque was assessed in the participants’ self-selected dominant limb using an isokinetic dynamometer (Cybex Norm, Cybex International, New York, NY, USA) at three different ankle angles (10°, 0°/neutral angle and -5°) and one isokinetic speed (60°·sec^-1^). Participants were seated in a supine position with a hip angle of 85° and their self-selected dominant leg fully extended. The dominant foot was secured to the footplate of the dynamometer using fixed unyielding straps, whilst ensuring the lateral malleolus was aligned with the center of rotation of the dynamometer. Participants were additionally strapped at the hip, distal thigh and chest with fixed unyielding straps to minimize synergistic movement that may unintentionally alter force production from the desired muscle group. Prior to undertaking any MVCs, the participants completed a series of 4–8 warm-up PF and DF contractions at 50% of their self-perceived maximal effort. Participants subsequently performed four isometric MVC first with PF (×2) and then DF (×2) at each of the three ankle angles and one isokinetic speed, with 2 minutes of rest between contractions. The order of the contractions was randomized for each participant, with the first angle being repeated at the end to ensure adequate warm-up and to diminish any learning effect and/or development of fatigue between contractions. MVCs were repeated if there was >10% difference between MVCs to ensure participants’ true MVC was obtained for each angle. The highest recorded PF and DF MVCs were used for subsequent analysis. Verbal encouragement and on-screen biofeedback were provided during each effort.

### EMG and RTD as potential indirect fiber type indices

In view of such potentially impactful research on post-menopausal women, the issue of methodology may sometimes be limiting, especially with invasive laboratory protocols. The majority of *in vivo* human studies commenting on muscle fiber composition of muscles have utilized biopsy material to determine myosin heavy chain composition (through myosin adenosine triphosphatase/mATPase enzyme activity) and hence classify a muscle as fast, intermediate or slow. By its nature however, this methodology is invasive. Non-invasive ways to assess fiber type composition have been developed, such as tensiomyography [25] and electromyography/EMG [26]. When action potentials travel along the sarcolemma of muscle fibers to simulate these into a contraction, they create a myoelectric signal (intensity and pattern) the sum of which is captivated using either intramuscular-iEMG or surface-sEMG. It is understood that sEMG power spectrum shifts towards lower frequencies during isometric contractions [27]. This shift is similar to observations using iEMG, as such they confirm the validity of sEMG data as representative of muscle activation. Using isolated rat muscles for instance, it has systematically been demonstrated that muscles with a high proportion of fast glycolytic or with fast oxidative fibers have a higher sEMG signal as quantified by the mean power frequency (mPowerF) [26]. This observation demonstrates that sEMG, especially that recorded during contractions, can reliably and singularly be linked to the muscle’s fiber type composition (as well as muscle fiber cross-sectional area- aCSA) [26]. The potency of the sEMG signal to provide information on muscle fiber type composition is also supported by authors who propose and/or demonstrate that free-living EMG data are to be incorporated towards the development of externally valid mathematical models of intrinsic skeletal muscle properties [28, 29]. More recently thus, human work has also been conducted which corroborates the fact that the electrical signal of muscle is a strong signature for its fiber type composition [26, 30–32].

RTD development was calculated using the highest MVC at 0°, then utilizing the slope of the torque curve from the onset of contraction at intervals of 0-50ms, 0-100ms, 0-150ms and 0-200ms.

sEMG (using pre-gelled unipolar Ag-AgCl electrodes (Medicost, Denmark)) was used to assess muscle activation from the mean power frequency (mPowerF) of the GM and TA and additionally muscle co-contraction of the TA during PF MVC at the three designated angles. Two sEMG electrodes (skin contact size 30 mm×22 mm) were placed proximally at one third of the GM and TA muscle length, mid muscle belly, with a 1–2-mm gap between each electrode. Two reference electrodes (Medicost, Denmark) were placed on the head of the fibula and medial tibial condyle. Raw EMG was then recorded at 2,000 Hz, with the band pass filter set at 10–500 Hz and notch at 50 Hz. Calculation of co-activation was then utilized to quantify participants’ true PF MVC accounting for co-contraction.

### Muscle activation, co-activation and net MVC

Calculation of muscle co-activation (%) at 10°, 0° and -5° ankle angle was conducted utilizing the raw EMG signal (computed as root mean square (RMS) 500 ms either side of the instantaneous peak torque) of the TA during PF MVC divided by sEMG during DF MVC. Co-contraction torque was the product of percent co-contraction and maximal DF torque, assuming that the DF EMG/torque relationship is linear as demonstrated by previous authors [33]. Hence, net PF MVC torque was calculated as the sum of observed maximal PF torque and co-contraction torque.

*Net MVC* = *PF MVC torque* + *DF co-contraction torque*

mPowerF of the raw sEMG signal using Fast Fourier Transform (AcqKnowledge software (version 4.2; Biopac-Systems, Goleta, CA) was obtained over 1 second prior to PF and DF MVC, during MVC and post MVC at 0° (neutral) ankle angle. Within the study, based on the rationale detailed at the beginning of this section, a higher mPowerF was proposed to potentially be indicative of a greater proportion of type II fiber composition within the working muscle.

### Gastrocnemius medialis muscle volume

Calculation of GM muscle volume was undertaken through the assembly of anatomical cross-sectional areas at 25, 50 and 75% of resting GM length of participants’ dominant leg using B-mode ultrasonography (AU5 Harmonic, Esaote Biomedica). Detailed description of the methodology is reported in [34]. All scans and measurements were undertaken by the same investigator. The assessment of GM muscle volume utilized the truncated cone method which has previously been shown to have high reliability [34], and strong agreement with MRI-obtained values [35]. Correction for intramuscular adipose tissue (IMAT) within the GM Muscle Volume was undertaken using data reported by Csapo et al. [36], where young women were shown to have 4.6%, whilst older women exhibited 8.1% IMAT. Thus:

*Muscle Quality (uncorrected)* = *(peak PF MVC torque* + *DF co-contraction torque)* ÷ *Muscle Volume (uncorrected for IMAT)*

*Muscle Quality (corrected)* = *(peak PF MVC torque* + *DF co-contraction torque)* ÷ *Muscle Volume (corrected for IMAT*)

Where peak torque is the highest recorded MVC over 10°, 0° and -5°, corrected for co-contraction, and divided by muscle volume, the latter corrected for standard IMAT value. For uncorrected muscle, the same calculation applied though it utilized the raw (i.e. uncorrected) peak torque data, and the raw muscle volume data.

### Serum estradiol and tri-iodo-thyronine concentration

Estradiol (R&D Systems, Minneapolis, USA) and T3 (Abbexa Ltd., Cambridge UK) serum concentrations were measured using enzyme-linked immunosorbent assay (ELISA), following the manufacturer’s instructions. Estradiol assay sensitivity reported by the manufacturer is 12.1 pg/mL, range 12.3–3,000 pg/mL, Minimum Detectable Dose 4.84 pg/ml, with an intra-assay precision of 5.97% coefficient of variation (CV) and an inter-assay precision of 7.13%. T3 assay sensitivity reported by the manufacturer is < 35 pg/mL, range 78 pg/mL—5000 pg/mL, with an intra-assay precision <7.2% CV and an inter-assay precision at <10.1% CV. Serum samples were run in duplicate and in our hands CVs of 5.7% for E2 and 4.1% for T3 were attained. The mean of each sample was taken as the hormone concentration. Samples were then analyzed using an EL808™ Absorbance Microplate Reader (BioTek, Vermont, US) and software Gen5 (version 1.11; BioTek, Vermont, US). There was insufficient serum volume for 13 participants when undertaking analysis of T3 resulting in a sample size of 48 for the T3 data.

### Statistical analyses

Statistical analyses were carried out using SPSS (Version 22, SPSS Inc., Chicago, IL, USA). Parametricity was determined through both the Kolmogorov-Smirnov test (Whole sample) and Shapiro–Wilk test (premenopausal, postmenopausal (No_HRT) postmenopausal (Used_HRT)) testing normality of the data. Assessment of equality of variances between groups was conducted by the Levene’s test. If parametric assumptions were met, between group differences were examined by a one-factor ANOVA (pre-menopause, postmenopausal No_HRT and Used_HRT) with post-hoc pairwise comparisons conducted using the Bonferroni correction. However, if parametric assumptions were breached, between group differences were examined by a Kruskal-Wallis non-parametric ANOVA (pre-menopause, postmenopausal No_HRT and Used_HRT) with post-hoc pairwise comparisons being examined by Mann-Whitney U test. Pearson (or Spearman rank order for non-parametric data sets) correlations were used to define any associations between skeletal muscle functional properties vs. E2, T3 levels and E2:T3 ratio. Co-variates were considered total fat mass, BMI, total body mass or age with the confounding variable status ascertained through correlation analysis against the outcome measure of interest. Partial correlations, were carried out to control for body composition or age, to allow ‘hormones only effects’ to be discerned. Data are reported as Mean±SD and statistical significance was accepted when *P*≤0.05. Study power (β) and effect size (ɳ_p_^2^) are also reported. Statistical trends indicate 0.05>p<0.1.

## Results

### Descriptive characteristics of participants

Table 1 displays participants’ characteristics, segregated by participants’ estrogen background (pre vs post-menopause phase and HRT history). As expected, a Kruskal Wallis non-parametric ANOVA revealed a significant difference in age between pre vs post-menopausal women (*P*<0.001), but none between postmenopausal No-HRT vs Used_HRT (i.e. post-menopausal women were older than their pre-menopausal counterparts, but the two post-menopausal groups were of similar age). Another difference was where pre-menopausal women had a lower body fat percentage than both post-menopausal groups, whilst the two post-menopausal groups did not differ (*P*<0.001). With total fat mass, on the other hand, pre-menopausal women only exhibited a significant lower value compared to the Used_HRT (*P* = 0.039); whilst again, the two post-menopausal groups did not significant differ from one another. In other parameters, the three groups (pre-menopausal, No_HRT and Used_HRT) were well-matched with no statistical differences in height, body mass, BMI, lean mass, or physical activity scores (work, sport, leisure and global scores) between the groups (Table 1).

**Table 1:** Descriptive participant and hormone characteristics grouped by menopause phase and use of hormone replacement therapy (HRT).

		Post-Menopausal	
	Pre-Menopausal (*n* = 33)	No HRT (*n* = 12)	Used HRT (*n* = 16)
**Participant Characteristics**			
Age (years)	27 ± 11 ^a^	69 ± 6 ^b^	65 ± 7 ^b^
Height (cm)	166 ± 8 ^a^	161 ± 4 ^a^	161 ± 5 ^a^
Body Mass (kg)	70 ± 21 ^a^	71 ± 12 ^a^	72 ± 12 ^a^
BMI (kg/m^2^)	25.6 ± 7.3 ^a^	27.4 ± 4.6 ^a^	27.6 ± 4.4 ^a^
Body Fat (%)	33.8 ± 5.2 ^a^	42.1 ± 4.7 ^b^	42.3 ± 5.1. ^b^
Fat Mass (kg)	24.7 ± 13.7 ^a^	29.5 ± 7.7 ^ab^	29.9 ± 8.2 ^b^
Lean Mass (kg)	41.8 ± 8.0 ^a^	37.6 ± 4.9 ^a^	37.5 ± 4.3 ^a^
ASM (kg)	17.0 ± 3.4 ^a^	16.1 ± 3.2 ^a^	17.8 ± 4.1 ^a^
ASM/Height^2^ (kg/m^2^)	6.2 ± 1.1 ^a^	6.2 ± 1.1 ^a^	6.7 ± 1.3 ^a^
No of Contractive Pill users	16/33	5/12	12/16
Average years of Pill use	2.5 ± 3.9 ^a^	5.4 ± 8.3 ^ab^	7.1 ± 6.8 ^b^
Menopause Age (yrs)	N/A	51 ± 4 ^a^	47 ± 6 ^a^
Average years of HRT use	N/A	N/A	7.8 ± 5.2
Average years since HRT use	N/A	N/A	10.6 ± 8.9
**Physical Activity Scores**			
Work	2.38 (0.69) ^a^	2.44 (0.34) ^a^	2.69 (0.34) ^a^
Sport	2.75 (1.00) ^a^	2.50 (1.50) ^a^	2.25 (0.25) ^a^
Leisure	2.75 (0.88) ^a^	3.25 (0.94) ^a^	2.75 (0.75) ^a^
Global	7.75 (1.50) ^a^	8.38 (0.97) ^a^	7.50 (0.84) ^a^

Participant Characteristics values are means ± SDs. Physical Activity Score values are median (interquartile range). Labelled menopause/HRT status means in a row without a common letter differ, P < 0.05. ASM, Appendicular Skeletal Muscle Mass

Starting with Estradiol, unsurprisingly a Kruskal Wallis non-parametric ANOVA revealed a main effect of menopause phase/HRT (*P*<0.001). Pairwise comparisons revealed pre-menopausal participants to have 293% (*P*<0.001) and 315% (*P*<0.001) greater Estradiol concentration than their post-menopausal No_HRT and HRT_user counterparts (**Table 2**), with no difference in Estradiol between the two post-menopausal subsets (p>0.05).

**Table 2:** Serum estradiol and tri-iodo-thyronine concentrations classified by menopause phase and use of hormone replacement therapy (HRT).

		Post-Menopausal	
	Pre-Menopausal (*n* = 33)	No HRT (*n* = 12)	Used HRT (*n* = 16)
**Hormone Levels**			
Estradiol (E2) pg/ml	191 ± 31 ^a^	49 ± 13 ^b^	46 ± 8 ^b^
Tri-iodo-thyronine (T3) ng/dl	157 ± 92 ^a^	135 ± 53 ^a^	182 ± 129 ^a^
E2/T3 ratio	1.42 ± 0.49 ^a^	0.42 ± 0.18 ^b^	0.33 ± 0.14 ^b^

Values are means ± SDs. Labelled menopause/HRT status means in a row without a common letter differ, P < 0.05.

Notably, there was no main effect of estrogen status on T3 levels as noted in Table 2. Still, there was a trend for pre-menopausal participants to have -14% (*P*<0.1) lower T3 than Used_HRT, and conversely 17% (*P*<0.1) greater T3 than No_HRT, and for Used_HRT to have 35% higher T3 than the No_HRT group (Table 2).

The large Estradiol group differences were mirrored by E2:T3 ratio as a one factor ANOVA revealed a main effect of menopause phase/HRT use (*P* = 0.001; ɳ_p_^2^ = 0.654; β = 1.000). Pairwise comparisons revealed pre-menopausal participants to have 237% (*P*<0.001) and 334% (*P*<0.046) greater E2:T3 ratio than both their post-menopausal No_HRT and HRT_user counterparts (Table 2).

### Co-variates identification

**Table 3** illustrates associations between measures of body composition and age against a series of skeletal muscle structural and functional characteristics and hormone levels to identify potential co-variates. A spearman *rho* correlation revealed BMI to be positively correlated with isokinetic DF MVC (*rho* = 0.29; *P* = 0.021) and peak DF MVC (*rho* = 0.29; *P* = 0.024), but negatively with muscle quality corrected (*rho* = -0.36; *P* = 0.008). Total fat mass was positively correlated with isokinetic DF MVC (*r* = 0.26; *P* = 0.044), peak isometric DF MVC (*rho* = 0.30; *P* = 0.019) and PF net MVC 0° (*r* = 0.27; *P* = 0.036), but negatively with muscle quality corrected (*rho* = -0.36; *P* = 0.008) and E2:T3 ratio (*rho* = -0.33; *P* = 0.024). Body fat percentage was negatively correlated with corrected muscle quality (*rho* = -0.39; *P* = 0.003), Estradiol (*rho* = -0.31; *P* = 0.016) and E2:T3 ratio (*rho* = -0.55; *P*<0.001). Age was negatively associated with Estradiol (*rho* = -0.773; *P*<0.001), E2:T3 (*rho* = -0.630; *P*<0.001), and lean mass (*rho* = -0.232; *P* = 0.036), but was positively associated with fat mass (*rho* = 0.336; *P* = 0.008), percent body fat (*rho* = 0.484; *P*<0.001) and BMI (*rho* = 0.295; *P* = 0.021). Data for muscle quality (both uncorrected and corrected) is shown in Table 3.

**Table 3:** Bivariate and partial (correcting for age) correlations between measures of body composition, skeletal muscle characteristics and hormone levels where co-variates are identified.

	Plantar Flexion					Dorsi Flexion										
	Maximum Voluntary Contraction										Muscle Characteristics			Hormone Levels		
	0 deg	10 deg	-5 deg	Peak TRQ	isokin	0 deg	10 deg	-5 deg	Peak TRQ	isokin	MV	MV (IMAT)	MQ (IMAT)	E2	T3	E2:T3
**Bivariate Correlations**																
BMI (kg/m^2^)	0.20	0.11	0.17	0.18	0.19	0.25	0.25	0.03	0.29*	0.29*	0.47***	0.44**	-0.36**	-0.10	-0.05	-0.20
Body Fat (%)	-0.10	-0.18	-0.13	-0.12	-0.13	0.09	0.06	-0.02	0.12	0.03	0.25	0.21	-0.48***	-0.31*	0.16	-0.55***
Fat Mass (kg)	0.14	0.06	0.10	0.12	0.12	0.24	0.25	0.04	0.30*	0.26*	0.42**	0.39**	-0.36**	-0.13	0.05	-0.33*
Age (years)	-0.50***	-0.53***	-0.49***	-0.51***	-0.69***	-0.24	-0.28*	-0.23	-0.25	-0.42**	-0.05	-0.10	-0.43**	-0.77***	-0.23	-0.63***
**Partial Correlations (age)**																
BMI (kg/m^2^)	0.41**	0.33*	0.39**	0.40**	0.57***	0.35**	0.37**	0.11	0.39**	0.48***	0.52***	0.51***	-0.20	0.21	0.03	-0.00
Body Fat (%)	0.19	0.10	0.15	0.16	0.33*	0.24	0.24	0.12	0.29*	0.36**	0.33*	0.31*	-0.22	0.12	0.33*	-0.33*
Fat Mass (kg)	0.38**	0.30*	0.34**	0.36**	0.51***	0.35**	0.38**	0.14	0.42**	0.48***	0.48***	0.47***	-0.23	0.21	0.15	-0.11

Significant correlations are highlighted by

* p<0.05

** p<0.01

*** p<0.001.

Pearson correlations are highlighted in grey. Abbreviations: deg, degree; E2, Estradiol; isokin, isokinetic; MQ, muscle quality; MV, muscle volume; muscle volume corrected for intramuscular adipose tissue; MQ (IMAT), muscle quality corrected for intramuscular adipose tissue; Opt, optimum; T3, Tri-iodo-thyronine; TRQ, torque.

### Musculoskeletal characteristics

#### *(a)* Maximum PF strength

**Fig 1:**
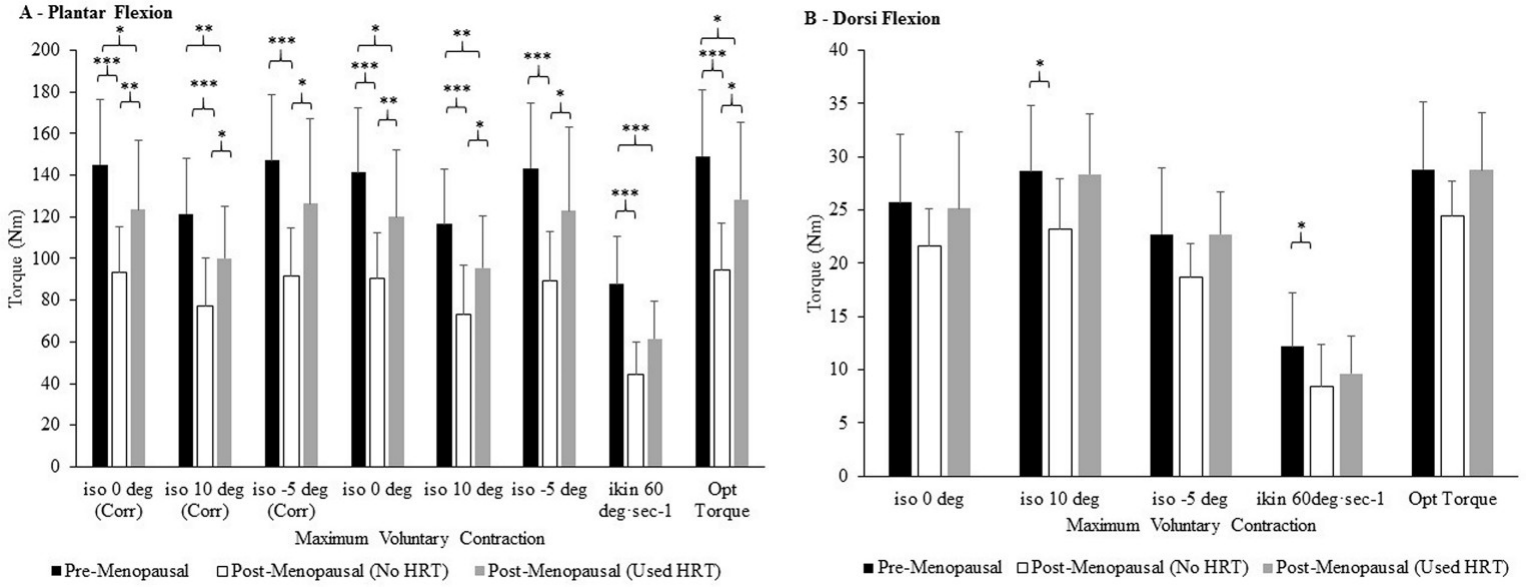
Comparison of participants’ plantar flexion (A) and dorsi flexion (B) maximum voluntary contraction at various isometric ankle angles (0, 10–5 degrees) and one isokinetic speed (60 deg·sec^-1^) categorized by participants’ menopause phase and use of hormone replacement therapy. Values are means ± SDs. Different from Pre-Menopausal and Post-Menopausal, * P < 0.05, ** P < 0.01, *** P < 0.001. Abbreviations: Corr, is MVC Corrected for co-contraction. Also referred to Net MVC in the text; Deg, Degree; ikin, isokinetic; iso, isometric.

When categorizing the females by menopause phase and splitting postmenopausal participants by their use of HRT following the menopause, the results revealed interesting outcomes as noted in **Fig 1**. A Kruskal Wallis non-parametric ANOVA revealed a main effect of menopause phase/HRT use on corrected 0° MVC (P<0.001). Pairwise comparisons revealed pre-menopausal participants to have 55% (*P*<0.001) and 17% (*P* = 0.016) greater corrected PF 0° MVC than their No_HRT and Used_HRT. In addition, Used_HRT had 32% (*P* = 0.006) greater 0° MVC than their No_HRT counterparts (Fig 1A). This pattern was similar for PF 10° MVC, as a Kruskal Wallis non-parametric ANOVA revealed a main effect of menopause phase/HRT use on corrected 10° MVC (*P*<0.001). Pairwise comparisons revealed pre-menopausal participants to have 58% (*P*<0.001) and 21% (*P* = 0.006) greater corrected PF 10° MVC than their No_HRT and Used_HRT counterparts. In addition, Used_HRT had 30% (*P* = 0.029) greater PF 10° MVC than their No_HRT counterparts (Fig 1A). At PF -5° MVC, a one factor ANOVA revealed a main effect of menopause phase/HRT use (P<0.001; ɳ_p_^2^ = 0.299; β = 0.993). Pairwise comparisons revealed pre-menopausal participants to have 60% (*P*<0.001) greater corrected PF -5° MVC than their No_HRT counterparts. In addition, Used_HRT had 38% (*P* = 0.031) greater PF -5° MVC than their No_HRT counterparts (Fig 1A). This pattern was continued for PF uncorrected MVC torque as noted in Fig 1. A one factor ANOVA revealed a main effect of menopause phase/HRT use on isokinetic PF MVC at 60°·sec^-1^ (P<0.001; ɳ_p_^2^ = 0.435; β = 1.000). Pairwise comparisons revealed pre-menopausal participants to have 97% (*P*<0.001) and 42% (*P*<0.001) greater isokinetic PF MVC than their no_ HRT and Used_HRT counterparts. Finally, regarding PF optimal torque MVC, a Kruskal Wallis non-parametric ANOVA revealed a main effect of menopause phase/HRT use (P<0.001). Pairwise comparisons revealed pre-menopausal participants to have 58% (*P*<0.001) and 16% (*P* = 0.023) greater corrected PF optimal torque than their No_HRT and Used_HRT counterparts, yet Used_HRT had 35% (*P* = 0.011) greater optimal torque than their No_HRT (Fig 1A).

#### *(b)* Maximum DF strength

With regards to DF, a one factor ANOVA revealed a main effect of menopause phase/HRT use on isometric DF 10° MVC (*P* = 0.021; ɳ_p_^2^ = 0.125; β = 0.709). Pairwise comparisons revealed pre-menopausal participants to have 24% (*P* = 0.021) greater DF 10° MVC than their No_HRT counterparts (Fig 1B). In addition, a one factor ANOVA revealed a main effect of menopause phase/HRT use on isokinetic DF MVC at 60°·sec^-1^ (*P* = 0.029; ɳ_p_^2^ = 0.155; β = 0.665). Pairwise comparisons revealed pre-menopausal participants to have 44% (*P* = 0.049) greater isokinetic DF MVC than their post-menopausal No_HRT counterparts (Fig 1B).

#### *(c)* Muscle volume and quality

With regards to GM muscle volume (uncorrected for IMAT), a Kruskal Wallis non-parametric ANOVA revealed no effect of menopause phase/HRT use on GM muscle volume (*P* = 0.154) (see **Table 4**). This finding was mirrored when with regards to GM muscle volume corrected for IMAT (*P* = 0.092). However, a partial correlation between age and GM muscle volume (corrected for IMAT) and controlled for BMI, revealed a negative association (*rho* = -0.30; *P* = 0.031). Interestingly, assuming that the age related loss of muscle mass at the onset of the menopause (47yrs Used_HRT and 51yrs No_HRT) is linear, the slope equated to No_HRT participants losing 1.3% of muscle volume per year in comparison to only 0.5% loss per year in the Used_HRT group.

**Table 4:** Descriptive muscle indices grouped by menopause phase and use of hormone replacement therapy (HRT).

		Post-Menopausal	
	Pre-Menopausal (*n* = 33)	No HRT (*n* = 12)	Used HRT (*n* = 16)
**Muscle Indices**			
mPowerF Pre PF MVC (Hz)	192 ± 56	186 ± 25	187 ± 27
mPowerF During PF MVC (Hz)	252 ± 22	250 ± 20	233 ± 31
Δ mPowerF PF MVC (Hz)	60 ± 55	65 ± 34	47 ± 35
mPowerF Pre DF MVC (Hz)	289 ± 58	270 ± 49	274 ± 49
mPowerF During DF MVC (Hz)	255 ± 26	246 ± 24	250 ± 16
Δ mPowerF DF MVC (Hz)	-34 ± 60	-24 ± 51	-23 ± 50
GM Muscle Volume (cm^3^)	234 ± 82	185 ± 40	223 ± 65
GM Muscle Volume (Corrected for IMAT) (cm^3^)	224 ± 78	170 ± 36	205 ± 59

Values are means ± SDs. No differences were observed between menopause phase and use of HRT. Abbreviations: DF, Dorsi Flexion; GM; Gastrocnemius Medialis; IMAT, Intramuscular Adipose Tissue; mPowerF, mean power frequency; MVC, Maximum Voluntary Contraction; PF, Plantar Flexion

**Fig 2:**
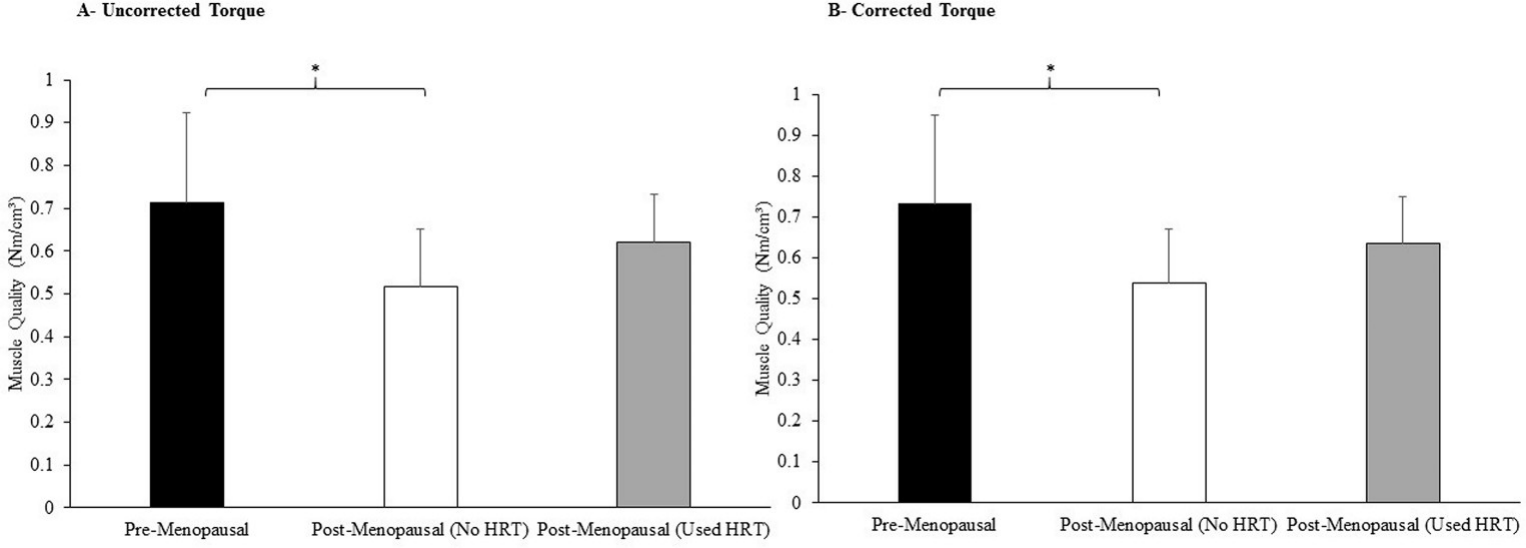
Comparison of participants’ muscle quality (utilizing uncorrected maximum torque (A) and maximum torque corrected for co-contraction (B) categorized by participants’ menopause phase and use of hormone replacement therapy. Values are means ± SDs. Different from Pre-Menopausal and Post-Menopausal, * P < 0.05.

Interestingly, a one factor ANOVA revealed a main effect of menopause phase/HRT use on muscle quality when utilizing uncorrected MVC torque (P = 0.013) (See Fig 2A). Pairwise comparisons revealed pre-menopausal participants to have 38% (P = 0.015) higher muscle quality than their post-menopausal No_HRT counterparts (Fig 2A), whilst there were no statistical differences between pre-menopausal and Used_HRT participants. This finding was mirrored when utilizing MVC torque corrected for co-contraction (P = 0.036; see Fig 2B), with pairwise comparisons revealing pre-menopausal participants to have 36% (P = 0.032) higher muscle quality than their post-menopausal No_HRT counterparts (Fig 2A), with again no statistical differences between pre-menopausal and Used_HRT participants.

#### *(d)* Potential indirect muscle fiber type indices: rate of torque development and mean power frequency)

**Fig 3:**
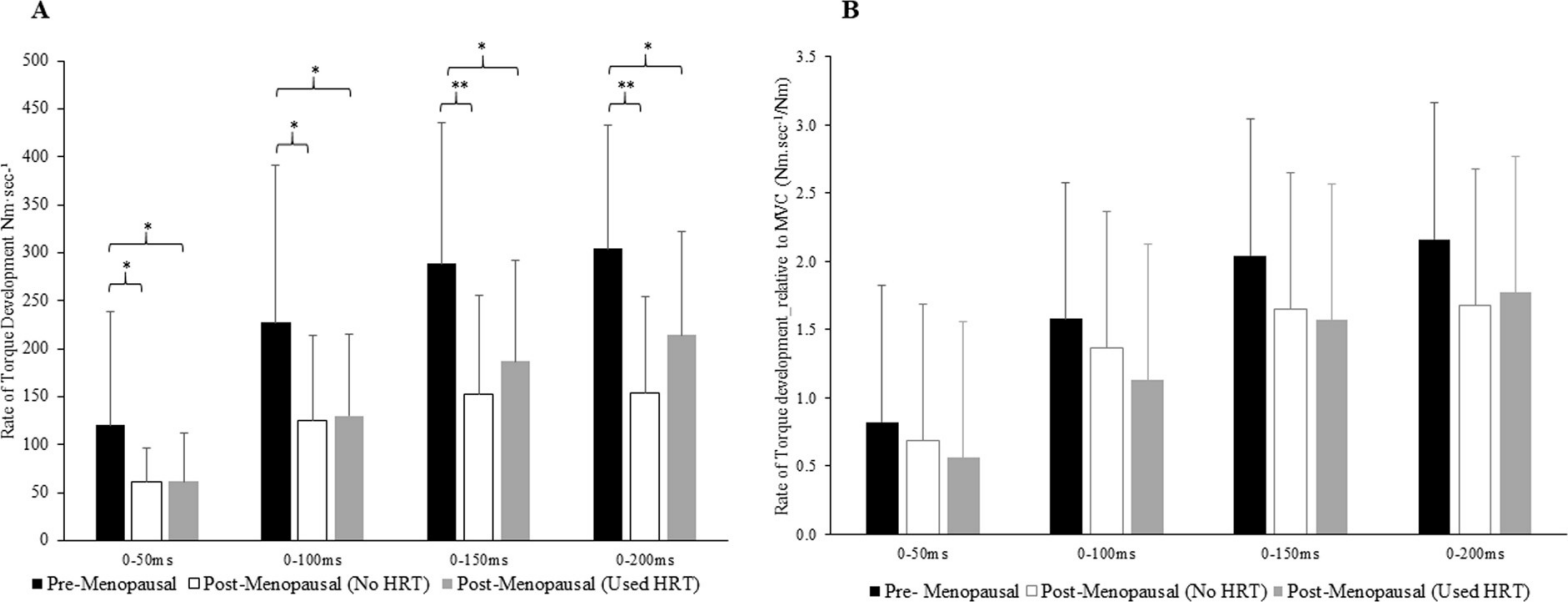
Comparison of participants’ plantar rate of torque development categorized by participants’ menopause phase and use of hormone replacement therapy. A) Absolute values. Values are means ± SDs. Different from Pre-Menopausal and Post-Menopausal, * P < 0.05, ** P < 0.01. **B. Relative values.** Values are RTD relative to each individual’s MVC. Different from Pre-Menopausal and Post-Menopausal.

**Fig 3** illustrates the effect of menopause phase and use/non-use of HRT on RTD. A Kruskal Wallis non-parametric ANOVA revealed a main effect of menopause phase/HRT use on PF RTD 0-50ms (*P* = 0.003). Pairwise comparisons revealed pre-menopausal participants to have 98% (*P* = 0.031) and 95% (*P* = 0.034) greater RTD 0-50ms than their post-menopausal no Used_HRT and No_HRT counterparts (Fig 3). Then for RTD 0-100ms, A Kruskal Wallis non-parametric ANOVA revealed a main effect of menopause phase/HRT use (*P* = 0.018). Pairwise comparisons revealed pre-menopausal participants to have 82% (*P* = 0.027) and 75% (*P* = 0.018) greater RTD 0-100ms than their post-menopausal No_HRT and HRT_user counterparts (Fig 3A). For RTD 0-150ms, a factor ANOVA revealed a main effect of menopause phase/HRT use (*P* = 0.003; ɳ_p_^2^ = 0.181; β = 0.888). Pairwise comparisons revealed pre-menopausal participants to have 90% (*P* = 0.008) and 55% (*P* = 0.036) greater RTD 0-150ms than their post-menopausal No_HRT and HRT_user counterparts (Fig 3A). Lastly for RTD 0-200ms, a one factor ANOVA revealed a main effect of menopause phase/HRT use (*P* = 0.001; ɳ_p_^2^ = 0.220; β = 0.951). Pairwise comparisons revealed pre-menopausal participants to have 98% (*P* = 0.001) and 42% (*P* = 0.046) greater RTD 0-200ms than their post-menopausal No_HRT and HRT_user counterparts (Fig 3A). There was, however, no significant difference in RTD when torque was expressed relative to MVC between groups (See Fig 3B).

**Table 4** illustrates the effect of menopause phase and use/non-use of HRT on mPowerF assessed during both PF and DF MVC. The PF mean mPowerF across the three groups at rest (~188±40Hz), and at MVC (~248±26Hz), i.e. ~35% relative increment from rest to MVC, showed no significant estrogen status effect. There were only trends for mPowerF in the resting PF to be greater than that of postmenopausal women, whilst at MVC the mPowerF of the No_HRT became closest to that of the premenopausal women with Used_HRT exhibiting the lowest values. In the dorsiflexors, the mean mPowerF at rest (~281±47Hz), and at MVC (~251±24Hz), i.e. ~ -9% relative decrement from rest to MVC, showed no significant estrogen status effect. Nonetheless mPowerF showed a trend for being highest in the premenopausal women at rest, during MVC and in terms of relative decrement from rest to MVC in the DF (see **Table 4**).

#### *(e)* Serum hormone associations against potential indirect muscle fiber type indices and muscle volume

In **Table 5** a series of bivariate correlations were run between serum Estradiol levels, T3 levels and the E2:T3 ratio against mPowerF of the *gastrocnemius medialis* during PF MVC, mPowerF of the *tibialis anterior* during DF MVC and the RTD during PF at 0° and muscle quality. Estradiol levels were positively correlated with RTD at 0-50ms (*rho* = 0.39; *P* = 0.002), 0-100ms (*rho* = 0.42; *P* = 0.001), 0-150ms (*rho* = 0.47; *P*<0.001), 0-200ms (*rho* = 0.48; *P*<0.001) and muscle quality (*rho* = 0.32; *P* = 0.019; Table 5). These positive correlations persisted even after controlling for age (see Table 5). In addition E2:T3 ratio was also positively correlated with RTD at 0-50ms (*rho* = 0.37; *P* = 0.010), 0-100ms (*rho* = 0.37; *P* = 0.010), 0-150ms (*rho* = 0.36; *P* = 0.012), 0-200ms (*rho* = 0.35; *P* = 0.016). Running correlations for Estradiol after controlling for body fat percentage (a previously identified covariate), the above-identified positive correlations were strengthened (RTD at 0-50ms (*rho* = 0.45; *P*<0.001), 0-100ms (*r* = 0.47; *P*<0.001), 0-150ms (*rho* = 0.50; *P*<0.001), 0-200ms (*rho* = 0.50; *P*<0.001). This was mirrored for E2:T3 ratio for RTD at 0-50ms (*rho* = 0.51; *P*<0.001), 0-100ms (*rho* = 0.46; *P* = 0.001), 0-150ms (*rho* = 0.40; *P* = 0.006) and 0-200ms (*rho* = 0.38; *P* = 0.008).

**Table 5:** Bivariate and partial (correcting for age) Spearman correlations between hormone levels against electromyography mean power frequency (pre, during, change) and rate of torque development.

	Plantar Flexion			Dorsi Flexion								
	Mean Power Frequency							Rate of Torque Development				
	Pre MVC	MVC	Δ mPowerF	Pre MVC	MVC	Δ mPowerF	0–50	0–100	0–150	0–200	MV (IMAT)	MQ (IMAT)
**Hormone Levels**												
Estradiol (E2) pg/ml	-0.05	0.24	0.12	0.11	0.05	-0.02	0.39**	0.42**	0.47***	0.48***	0.30*	0.27*
Tri-iodo-thyronine (T3) ng/dl	0.13	-0.13	0.22	0.19	-0.06	-0.154	0.06	0.09	0.15	0.16	-0.17	0.11
E2:T3 ratio	-0.07	0.06	0.05	-0.05	0.06	0.04	0.37*	0.37*	0.36*	0.35*	0.25	0.17
**Partial Correlation (age)**												
Estradiol (E2) pg/ml	-	-	-	-	-	-	0.40**	0.37**	0.33*	0.26*	0.35**	0.11
E2:T3 ratio	-	-	-	-	-	-	0.31*	0.25	0.16	0.09	0.30*	-0.22

Significant correlations are highlighted by

* p<0.05

** p<0.01

*** p<0.001.

Abbreviations: mPowerF, mean power frequency; MVC, maximum voluntary contraction; MQ (IMAT), muscle quality corrected for intramuscular adipose tissue; MV (IMAT), muscle volume corrected for intramuscular adipose tissue.

Finally, there were no significant correlations between Estradiol, T3 or E2:T3 ratio against GM muscle volume when uncorrected for IMAT. However, when GM muscle volume was adjusted for IMAT, there was a positive correlation between Estradiol and GM muscle volume (*rho* = 0.30; *P* = 0.027; Table 5). It should be noted that this association between Estradiol and GM muscle volume adjusted for IMAT, remained when controlling for age in the whole sample (*rho* = 0.35; *P* = 0.009; Table 5), and in the post-menopausal group sample (*rho* = 0.412; *P* = 0.040). However, the association was not present in the pre-menopausal group (*rho* = 0.125; *P* = 0.519).

## Discussion

Three key observations in the literature informed this study: (1) previous research links low thyroid levels with a conversion from fast to slow fiber types [37] (although females with low thyroid hormones have a higher amount of type II than their male hypothyroidism counterparts [20]); (2) low estrogen such as seen in the menopause is synonymous with decreased lean tissue content [38, 39], lower physical performance [40], and animal work corroborates with these observation [41, 42]; (3) skeletal muscle is an estrogen-responsive tissue as it contains receptors for this ligand [43–47]. Based on these observations, it followed that the interaction of Estradiol and Tri-iodo-thyronine, even in healthy individuals, would be of clinical interest. Our study therefore set out to elucidate a few of these point using a cross-sectional design. We anticipated that (1) Lower levels of estradiol (and/or no HRT history) is linked to decreased muscle function (decreased isometric MVC, isokinetic strength and muscle quality), (2) Ageing is associated with perceptible decrements in T3 levels thereby modulating muscle activation profile and hence potentially indicating fiber type percentage shift towards a slow phenotype (resulting in slower rate of torque development–RTD—and lower Mean Power Frequency–mPowerF-); (3) estradiol to Tri-iodo-thyronine ratio (i.e. E2:T3) is an endocrine modulator of skeletal muscle phenotype, (4) high body fat in the post-menopausal status alters muscle EMG profile and RTD (these being two proposed indirect indices of muscle fiber type), potentially through decreasing the fast-to-slow transition of fiber type in older women.

### Estrogen levels affect muscle function

Our data confirms the first hypothesis: Lower levels of estradiol (and/or no HRT history) is linked to decreased muscle function (decreased isometric MVC, isokinetic strength and muscle quality). Indeed post-menopausal No_HRT women had lower isometric, and isokinetic torque in both the plantarflexor and dorsiflexor muscle groups, compared to the post-menopausal Used_HRT, themselves having a lesser performance in these muscle strength outcome measures compared to pre-menopausal women. In parallel and as expected, the grouping comparison and the correlation between age and Estradiol were statistically significant (*rho* = -0.773, p<0.001). These findings are consistent with previous research comparing pre-menopausal to post-menopausal [40], or post-menopausal Used_HRT vs No_HRT [15, 16, 48]. Our current, results are even more striking when one considers that in the HRT group, therapy had been ceased 10.6 ± 8.9 years prior to the current study and the levels of estrogen were now within expected post-menopausal levels [49, 50]. The physiological pathway for the effect of estrogen on muscle mass and function is proposed to be anti-apoptotic [51] as well as through IGF-1, a potent stimulator of skeletal muscle growth and regeneration, which is found to be involved in the estrogen signal transduction pathway [52, 53]. In addition, human skeletal muscle cell culture work has demonstrated that estradiol and selective estrogen receptor modulator (SERM) treatments influence estrogen receptor co-regulator gene expression in human skeletal muscle cells [54]. Our data, which show better physiological reserves in previous HRT users, would thus suggest that these (cellular and molecular) effects of HRT, are maintained in the long term, even after cessation of the therapy.

### Thyroid hormone levels across the ages, estrogen levels and fiber type composition

Our second hypothesis was that ageing is associated with decrements in circulating T3 levels that will be associated with slow-to-fast fiber type transition (resulting in slower rate of torque development–RTD—and lower Mean Power Frequency–mPowerF-). In our sample of women, we did not see a systematic decrease in circulating T3 levels with menopause, but we observed a trend for Used_HRT to have in fact, higher T3 levels than pre-menopausal women. The grouping comparisons and the correlation between age and T3 revealed no association (*rho* = -0.228, p = 0.12) and a post hoc power calculation suggests a sample size of 23 participants in each group for future work. Notably also the grouping comparisons and the correlation between age and either E2:T3 ratio were statistically significant (*rho* = -0.630, p<0.001). These observations are somewhat in agreement with previous research in that whilst thyroid hormone levels are not necessarily found to alter with healthy ageing in animal models [1], in humans, however, there is a trend towards decreased T3 with ageing [55]. Some studies however show similarly to us, that ageing is not necessarily synonymous with T3 levels changes. In fact, it would seem that sex here is the factor and in women, free thyroid hormone levels are not changed with ageing [56].

The physiological pathway for the effect of thyroid on muscle mass and function is proposed, using microarray assays validated through RT-PCR, as being mediated via an upregulation of MDM2 protein which is an E3 ligase [57]. The researchers report that in fact T3 exclusively upregulates MDM2 in type I fibers. What’s more, any attempt at pharmacologically blocking MDM2 in cultured myocytes leads to a 35% increase in fiber atrophy. This therefore leads to a magnification of the effect of T3 (increasing its atrophying effects from 12% to 35%). What is also now understood, is that the dual and yet at times opposing effects of T3 are evident in skeletal muscle where it not only induces a slow-to-fast transition [21] but also increases mitochondrial content and myoglobin expression. The net outcome of this mitochondrial effect is the facilitation of increased aerobic capacity [58]. At the same time, T3 has been linked to an increase in protein breakdown [59, 60]. Conversely also, T3 has been linked to enhanced protein synthesis [61]. It is as yet unclear what conditions prevail for the overall result of these two opposing forces (catabolic vs anabolic), to ultimately result in a healthy vs a sarcopenic status. Certainly previous work, including ours, consistently shows a decrease in muscle tissue content with increased age, both in males [62] and females [24, 34], which would tend to support the idea that the sarcopenic pathway overrides any anabolic potential in the effects of T3.

It has been reported that estrogen and thyroid hormone may interact in the regulation of gene expression, and that thyroid hormone may increase the expression of estrogen receptors [63–65]. Therefore, our third hypothesis was that, estradiol to Tri-iodo-thyronine ratio (i.e. E2:T3) is an endocrine modulator of skeletal muscle phenotype. In terms of inferred indices of fiber type composition, our mPowerF data would tend to support the idea that the DF has a greater proportion of fast fibers than the PF [66], irrespective of estrogen status. Remarkably, with comparisons of RTD (at 0-50ms, 0-150ms and 0-200ms regions) in the PF as an indirect index of this muscle’s fiber type composition, we consistently found a main effect of estrogen level with differences following a staircase effect with pre-menopausal >> Used_HRT > No_HRT. These dissimilarities were of high magnitudes, ranging from 42–98% differences between pairs. As far as how these were associated with hormone levels and/or ratio, Estradiol and E2:T3 were significantly associated with RTD (in all regions) but T3 on its own was not singularly associated with any of the outcome variables. mPowerF was not associated with any hormone levels and/or E2:T3 ratio. Thus our data reach beyond our second hypothesis. Indeed, they suggest that all things being equal (i.e. only small group differences in T3 as seen in the present study cohort), the effects of high estrogen levels overshadow those of T3. Thus overall, estrogen influences a push towards greater RTD which is a potential indication of greater proportion of type II fiber type. It is timely here to also note that our observations cannot necessarily be assigned to the effects of Estradiol only. This assertion is on one main account: the above-described physiological pathway through which each of these hormone is known to affect skeletal muscle. Our data may suggest that the individual variations in E2:T3 point to a separate importance in this parameter, thereby supporting our third hypothesis. Indeed, we may have expected a loss of association between the hormones and the physiologic outcomes, had T3 data only contributed noise in the relationship.

Given that the physiology theorem is that during ramped contractions the motor units are recruited in an orderly fashion from slow to fast, it could also be surmised that the change in signal from rest to maximal activity may be yet another valuable indirect indicator of the muscle fiber phenotype. In the plantarflexors, EMG frequency increased by ~35%, whereas in the dorsiflexors it decreased by ~9% from rest to MVC. We thus infer that whilst in the PF muscle efforts occur with an increase in action potentials, the opposite is true for the dorsiflexors. However, there was no difference in muscle activation strategies between the three estradiol status groups again bringing into question the usefulness of this EMG measure as an independent indirect, non-invasive quantifier of muscle fiber type composition.

### The impact of adiposity on estrogen related muscle phenotype

Our fourth and final hypothesis was that high body fat in the post-menopausal status alters muscle EMG profile, and RTD, both proposed indirect indices of muscle fiber type (potentially through decreasing the fast-to-slow transition of fiber type) in older women. Total fat mass was positively correlated with isokinetic DF MVC and PF net MVC 0°, but negatively associated with muscle quality, E2:T3, and peak isometric DF MVC. In addition, body fat percentage was negatively correlated with muscle quality, Estradiol and E2:T3. These associations were in agreement with our predictions except for the surprising negative correlation between Estradiol and body fat percentage (*rho* = -0.306; *P* = 0.008) when we would have expected to see higher Estradiol in the more adipose participants [17]. However, given that HRT supplementation is previously reported to be associated with better body composition in post-menopausal women [67], our current results may be suggestive of the body composition improvement effects of Estradiol to supersede those of high adiposity. Notwithstanding these we observed, as predicted [24], a negative correlation between body fat percentage against muscle quality adjusted for IMAT (*rho* = -0.48; *P*<0.001). This observation would suggest that as muscle quality is high, so are Estradiol levels whilst both total body fat mass and relative percent body fat are low. In other words, estrogen however indirectly, is synonymous with better skeletal muscle phenotype outcome. The distinction of the effects of estrogen were also confirmed in the use of partial correlations, correcting for age. It is also notable that in the post-menopausal group, the relationship between estradiol and IMAT corrected muscle volume remained (rho = 0.412; P = 0.040). Thus, the effect we are describing cannot be said to simply be a factor of much higher levels of estrogen in the young.

Initially, we anticipated that adiposity may impact on muscle function on several levels, owing to a number of previous studies that suggest a link between adiposity and neural activation [24, 68]. Although increased adiposity was associated with decreased muscle quality in the present study, neither absolute fat mass nor %body fat were found to be associated with any of the proposed indirect indices of fiber type composition (mPowerF or RTD). This would mean that at least based on this sample of post-menopausal status segregated women, our fourth hypothesis which was that high body fat in the post-menopausal status alters muscle EMG profile, and RTD, both proposed indirect indices of muscle fiber type (potentially through decreasing the fast-to-slow transition of fiber type) in older women, was not supported.

### Some of the challenges of this in vivo human work

A potential source of error from our data is linked to the fact that EMG spectra are influenced by muscle fiber length, temperature, fatigue, as well as body composition [28]. We have now established that body composition (BMI and Body mass) did not significantly differ between the 3 groups. Also given the fact that the environment laboratory conditions were standardized, and fatigue was not allowed to build during the test protocol, it leaves only fascicle length which was not directly controlled. This parameter is affected by the anatomical characteristics of the individual as well as the stiffness of the tendon/aponeurosis to which the muscle is attached. Therefore, given the same ankle angle (which we did control for), the length of the fascicle could still have differed between participants thereby potentially introducing noise in our data. Moderating this criticism however, is that this is a standard biomechanics assessment practice such as seen with previous research which groups tendon properties by estrogen status [69, 70]; for a review see [71]). Thus, the mean data from the three groups would still be valid based on similar degree of relative lengthening/shortening of the muscle within each cohort.

Another potential confounding variable is that the effects of T3 are highly muscle dependent. In rats for instance, the effects of the hormone are more marked in the soleus muscle where it accelerates twitch parameters by up to 57% whilst decreasing the proportion of type I fibers by up to 77% in both young and old animals [3, 5]. The sensitivity to T3 is less so in the *extensor digitorum longus* (EDL) where it accelerates twitch parameters by only up to 24% and any effect is limited to older animals [1]. To somewhat account for differential sensitivities to T3, we analyzed two different muscles, PF and DF. Indeed the two are reported as having a different proportion of fiber types, with GM (representative of the PF) having ~50% Type I [72] vs *tibialis anterior* (TA; representative of the DF) having ~40% type I [66, 73, 74]. Therefore, the potential for a low T3-level mediated muscle activation profile, and presumably fiber type transition towards a slower phenotype, may have had greater scope to be more pronounced in the ‘faster’ muscle. Data from the TA, a relatively fast muscle, thus tackled this side of the issue and yet no singular effect of T3 was found. Therefore, we would recommend that future studies use other, more distinctly fast vs slow muscles, to further elucidate the impact of T3 and Estradiol, on muscle activation profile and potentially therefore, fiber-type transition.

Lastly, like the majority published research on muscle fiber types, we have expressed our indirect quantified fiber type as the fractions of fiber numbers. We acknowledge that this is a simplified binary (fast vs slow) fashion. However fiber types are seldom expressed as the fractions of cross-sectional area [75, 76], possibly owing to the complexity of assuming area and tissue density when intramuscular collagenous tissue and fatty infiltration can easily confound any calculation. Where studies have made a point of systematically quantifying exact CSA fraction for fiber types in different muscles [77], they have again confirmed (tough not always e.g. [6]) the sex dependence. For instance, in the *vastus lateralis*, it is reported that in terms of relative CSA, in males IIA > IIX > I, whereas in females I > IIA > IIX [78] thus lending support to the idea that sex has a major impact on muscle phenotype. At face value, this would suggest that estrogen has a ‘slowing’ effect on fiber type phenotype. This conclusion is diametrically opposed to our observation of a ‘speeding’ effect of estrogen on fiber type phenotype. It may be that either a) in a comparison of females to females, estrogen has a ‘speeding’ effect owing to the interplay of other hormones, b) based on estrogen receptor numbers and fiber type prevalence differences between muscle, the effects of estrogen are muscle specific, or c) the difference in muscle fiber prevalence in men compared to women is linked to one of the plethora of differences between the two sexes, not necessarily estrogen levels. In short, more work is required in this area.

Throughout, we have argued the fact that fiber type may be a key modulator of RTD. We should now also consider that RTD is also affected by tendon properties [79, 80], and that the RTD in terms of Nm·sec^-1^ is also, all else being the same, positively related to the maximal force the muscle can generate. Previous work has shown the impact of tendon on twitch parameters [79, 80] and plyometric performance [81, 82]. Given this, it may be that the age-related decrease in RTD that we have attributed to changes in fiber type (consistent with the animal models), may in fact be an effect of a decrease in tendon stiffness. However, the correlation between estradiol and RTD (even after correction for age), is at odds with the influence of tendon, where if anything, low estrogen is associated with a stiffer tendon (for a review, see [71]). Thus, our findings appear consistent with the suggestion that the decrease in RTD data is reflecting a fast-to-slow fiber type transition during aging, or perhaps an integral skeletal muscle structure effect [83, 84]. This conclusion corroborates with the work of previous researcher which suggests direct estrogen effect on myosin-actin coupling [85]. We should treat this conclusion with some caution, however, as the RTD normalized to MVC did not show a significant age-related decline, even though the pattern of change was in the same direction.

Perhaps one limitation of our study is that we did not assess the dose and duration, nor possible changes in diet and exercise since menopause, nor possible influences of alcohol consumption and smoking history.

In view of our findings, we recommend that future work uses biopsies to confirm or otherwise refute our current conclusions regarding the potential fiber type alterations. Though this may not necessarily have been much of an issue in our case, as we utilized a post-menopausal sample (hence minimal estrogen levels variations). We also recommend that blood samples are taken over a course of a few months to confirm the average estrogen (and/or thyroid hormone) levels of each woman.

The data is in this study indicate that the benefits of hormone replacement therapy for muscle function are long lasting even after cessation of said therapy. This may have significant implications as a limited duration of hormone replacement therapy will diminish the often reported risks of developing cancer and cardiovascular diseases [86] and hence may increase the number of women that can safely be prescribed short duration hormone replacement therapy.

## Conclusion

The current study showed a few striking results. First, not only do they confirm the physiological advantage of HRT use in terms of physical functions, they demonstrate for the first time that (assuming similarities of other potential cofounders in the intervening period between HRT use, cessation and study timelines e.g. habitual lifestyle and health history) these benefits persist years beyond termination of the therapy. In relation to this, we found that age and body fat percentages were modulators of estrogen levels and/or ultimately muscle size (especially when corrected for IMAT) and muscle quality (described as the force normalized to the muscle tissue content). Whether this association is causative remains to be elucidated. One reason adiposity may have a negative impact on muscle quality may be as it is commonly associated with low grade inflammation and oxidative stress. Also notable, was our observation that RTD (but not mPowerF) is associated with Estradiol level and E2:T3 ratio. This may therefore distinguish RTD as a useful and sensitively maker of estrogen/HRT history effect on skeletal muscle. Whether RTD proposed here as an indirect marker of *in vivo* muscle fiber composition, proves to be so, remains to be confirmed. Using this sensitive marker of muscle activation profile (and thus possibly fiber type phenotype), our data additionally suggest that high circulating estrogen, with unchanging T3 levels, could lead to a marked change in muscle activation profile, theoretically suggestive of higher proportion of type II fibers in a muscle.

## Supporting information

S1 Data (XLSX)
